# Transcription Factors Involved in Prostate Gland Adaptation to Androgen Deprivation

**DOI:** 10.1371/journal.pone.0097080

**Published:** 2014-06-02

**Authors:** Rafaela Rosa-Ribeiro, Umar Nishan, Ramon Oliveira Vidal, Guilherme Oliveira Barbosa, Leonardo Oliveira Reis, Carlos Lenz Cesar, Hernandes F. Carvalho

**Affiliations:** 1 Department of Structural and Functional Biology, State University of Campinas, Campinas, São Paulo, Brazil; 2 Laboratory of Bioinformatics, National Center for Research on Energy and Materials, Campinas, São Paulo, Brazil; 3 Division of Urology, State University of Campinas, Campinas, São Paulo, Brazil; 4 Department of Quantum Physics, State University of Campinas, Campinas, São Paulo, Brazil; 5 National Institute of Photonics Applied to Cell Biology (INFABiC), State University of Campinas, Campinas, São Paulo, Brazil; National Health Research Institutes, Taiwan

## Abstract

Androgens regulate prostate physiology, and exert their effects through the androgen receptor. We hypothesized that androgen deprivation needs additional transcription factors to orchestrate the changes taking place in the gland after castration and for the adaptation of the epithelial cells to the androgen-deprived environment, ultimately contributing to the origin of castration-resistant prostate cancer. This study was undertaken to identify transcription factors that regulate gene expression after androgen deprivation by castration (Cas). For the sake of comparison, we extended the analysis to the effects of administration of a high dose of 17β-estradiol (E2) and a combination of both (Cas+E2). We approached this by (i) identifying gene expression profiles and enrichment terms, and by searching for transcription factors in the derived regulatory pathways; and (ii) by determining the density of putative transcription factor binding sites in the proximal promoter of the 10 most up- or down-regulated genes in each experimental group in comparison to the controls *Gapdh* and *Tbp7*. Filtering and validation confirmed the expression and localized EVI1 (Mecom), NFY, ELK1, GATA2, MYBL1, MYBL2, and NFkB family members (NFkB1, NFkB2, REL, RELA and RELB) in the epithelial and/or stromal cells. These transcription factors represent major regulators of epithelial cell survival and immaturity as well as an adaptation of the gland as an immune barrier in the absence of functional stimulation by androgens. *Elk1* was expressed in smooth muscle cells and was up-regulated after day 4. *Evi1* and *Nfy* genes are expressed in both epithelium and stroma, but were apparently not affected by androgen deprivation.

## Introduction

Androgens are required for prostate development, growth and physiology, by activating the androgen receptor (AR), which is expressed in both epithelial and stromal cells of the adult prostate gland [1], [2]. More than 300 proteins have been identified to contribute to AR activation and to modulate its transcription activity [3]–[6], to promote a variety of gene expression patterns in cells and tissues [7].

Understanding the mechanisms of androgen regulation in the prostate gland is important, because the prostate is affected by several different diseases, in particular prostate cancer (PCa). Several ways exist to treat prostate cancer and promote cell cycle arrest and/or epithelial cell death. Treatments involving androgen manipulation include surgical castration (bilateral orchiectomy), antiandrogens (usually AR antagonists), or substances that inhibit androgen synthesis (5α-reductase inhibitors, gonadotrophin-releasing hormone blockers) [8]. 17β-estradiol exerts anti-androgen effects by blocking the hypothalamic production of gonadotropin-releasing hormone and thereby inhibiting the production of testosterone by the testes [9], but also acts locally via interactions with either of the estrogen receptors found in the gland.

The two major drawbacks to the use of antiandrogens or androgen deprivation therapies are the systemic side effects, including physiological (bone loss, muscle weakness, temperature deregulation, cardiovascular problems) and behavioral changes (loss of stamina, apathy, loss of libido and depression) on the one hand, and progression to castration-resistant prostate cancer (CRPC), which is more aggressive than the original disease, on the other.

Although androgens are highly important for prostate cancer development, after androgen deprivation the disease progresses to a castration-resistant state that may be driven by AR mutations, amplifications and/or ligand-independent activation, which can keep the prostate epithelial cells alive in an androgen-poor environment [10], [11]. In addition to the mechanisms centered on AR expression and functioning, a variety of chromosomal and physiological changes are associated with PCa progression (i.e. tumor growth, metastasis and androgen independence) [12], and chromosome aberrations, including frequent bridging (chromoplexy) [13].

Previous analyses of gene expression revealed significant aspects of prostate physiology [14]–[19]. These studies employed different strategies to obtain the data, and arrived at different subsets of genes that are differentially expressed in response to challenging hormonal conditions. Given the extreme drop in secretory function in response to androgen, and the complex interactions between the epithelium and the stroma, it is possible that subtle changes in physiologically important factors are obscured in the mass of information obtained. For instance, Desai et al. (2004) pointed out a progressive increase in PTEN expression in the epithelial cells and several genes grouped together to characterize an “immune-inflammatory” response, which was validated and correlated with a high concentration of immune-system cells including macrophages, mast cells and lymphocytes [15]. The concentration of these cells is another complicating component in the analyses of gene expression, because they contribute their own mRNA. Asivartham et al. (2006) worked with isolated cells in primary cultures, but in these conditions, the contribution of mutual stromal-epithelial interactions is absent [19].

We therefore hypothesized that a better understanding of the nature of the cells that survive castration would benefit the search for strategies to allow a blockade or at least an extension of the time needed for the transition to the CRPC, and that the identification of regulatory networks for transcription factors (TF) could reveal new therapeutic targets.

Pursuing the idea that additional TF could be co-opted for coordinating gene expression that would contribute not only to epithelial-cell death but also to an adaptation of the organ in general and the epithelium in particular to varying hormonal conditions, notably complete androgen deprivation, and that these changes increase the susceptibility to progression to CRPC, we performed gene expression profiling using DNA microarrays to identify TF associated with the most-regulated genes after androgen deprivation by surgical castration (Cas group). We included in the analyses a group of rats that received a high dose of 17β-estradiol (E2 group) (falling androgen level and high estrogen) and a group of rats that were castrated and treated with E2 (Cas+E2 group; low androgen, high estrogen).

Inspired by the study of Yeh et al. (2009), we attempted to identify regulatory networks among the genes obtained from microarray data, by examining the relatedness between the regulated genes and structural signatures in their promoters [20]. In a first approach, we identified all genes showing differential expression in each experimental group when compared to the controls. The differentially expressed genes were arranged into enrichment terms, and the resulting regulatory gene networks constructed were used for the identification of candidate TF. In a second approach, we examined the 3,000 bp proximal promoter of the ten most differentially expressed genes for the presence of putative transcription-factor binding sites, and determined their relative abundance with respect to the corresponding promoter regions of two internal control genes (i.e. not regulated by each treatment). The filtered TF were then validated by qRT-PCR and localized in the gland by immunohistochemistry. The expression pattern and tissue location of these TF appear to be important for the fine-tuning of prostate adaptation to the androgen-deprived environment.

## Material and Methods

### Animal Treatments

Forty-eight 21-day-old male Wistar rats were obtained from the Multidisciplinary Center for Biological Research (CEMIB), University of Campinas. The animals were kept under normal light conditions (12-h light:dark cycle) and received filtered tap water and Purina rodent chow *ad libitum*.

On the 90^th^ day after birth, the rats were divided in four groups (n = 3) and assigned to different treatment groups. To cause androgen deprivation, we utilized three different procedures with different effects on epithelial cell apoptosis [21]. Animals in the first group were castrated (Cas) by orchiectomy via scrotal incision under ketamine (150 mg/Kg body weight) and xylazin (10 mg/kg body weight) anesthesia. Animals in the second group received a 25 mg/Kg body weight dose of 17β-estradiol diluted in corn oil (E2 group). The third group received a combination of both treatments (Cas+E2 group) (combined orchiectomy and 17β-estradiol). In the control group (Ct; normal androgen and estrogen), the animals received only the vehicle. Three days after the treatments, the rats were killed by anesthetic overdose, and the ventral prostate was dissected out for the microarray and immunohistochemistry analyses. For evaluation of the TF expression after castration, 24 animals were distributed in eight groups: one non-castrated (NC) and seven castrated (Cas) groups (1 to 7 days after surgery).

The procedures were approved by the Committee for Ethics in Use of Animals (CEUA) for the Institute of Biology, State University of Campinas (protocol nos. 1945-1 and 3000-1).

### RNA Extraction

Ventral prostates were dissected under RNAse-free conditions. Thirty mg of the tissue was used for total RNA extraction. Subsequently, the tissue fragments were extracted using Illustra RNAspin Mini kits (GE Healthcare, Buckinghamshire, UK) according to the manufacturer’s instructions. RNA purity was analyzed by the ratio of absorbance at 260/280 nm (values higher than 1.8) and by electrophoresis on 1.2% denaturing agarose gel. The RNA concentration in each sample was determined in an Ultrospec 2100 pro spectrophotometer (Amersham Biosciences, Cambridge, England).

### Microarray Hybridization

Whole transcript microarrays (Gene Chip Rat Gene 1.0 ST Array) purchased from Affymetrix (Santa Clara, CA, USA) were used for gene expression analysis. Microarray probes were synthesized from 500 ng of total RNA using a WT Expression kit (Ambion, Austin, TX, USA) according to the manufacturer’s instructions. Single-strand cDNA was synthesized containing a T7 promoter sequence, and the second-strand cDNA was synthesized by DNA polymerase in the presence of RNase H.

The antisense cRNA was synthesized and amplified by *in vitro* transcription (IVT) of the second-strand cDNA template using T7RNA polymerase. The cRNA obtained was purified to improve the stability of the cRNA. From 10 µg of purified cRNA the sense-strand cDNA (ss-cDNA) was synthesized by reverse transcription using random primers, and the ss-cDNA contained dUTP at a fixed ratio relative to dTTP. Then, the cRNA was degraded by RNase H and the ss-cDNA was purified.

In the second part of the protocol, 5.5 ng of ss-cDNA in a 31.2 µL volume was nicked and labeled using the GeneChip WT Terminal Labeling Kit (Affymetrix) according to the manufacturer’s instructions. The efficiency of the labeling procedure was controlled by a protocol that prevents hybridizing poorly labeled targets onto the probe array. The addition of biotin residues was checked in a gel-shift assay using 4% agarose gel in TBE buffer and monitoring for the presence of fragments with 200 bp or less.

For the hybridization, washing and staining steps, the GeneChip Hybridization, Wash and Stain Kit (Affymetrix) was used. 80 µL of the fragmented and labeled ss-cDNA solution was loaded on the gene chip probe array and incubated at 45°C, 60 rpm for 18 h. The hybridized chip was set in the fluid station to stain and wash (GeneChip Fluidics Station 450; Affymetrix), and scanned using the Affymetrix GeneChip Operating Software v1.3 program. The data were normalized and summarized by Expression Console software (Affymetrix), and the results were analyzed by bioinformatics.

### Analysis of Microarray Results

The expression profile was evaluated for biological replicates from the treatment and control groups. The summarized data from microarray experiment were processed using bioconductor R package (limma and affy libraries), the background correction was done by using RMA (Robust Multi-array Average) and the normalization was done by quantiles. The p-values were adjusted by FDR multiple test correction. Probe to gene annotation was done using the Affymetrix annotation files. Genes with fold-change (Log2) >1.5 or < −1.5 and adjusted p-value <0.01 were applied for subsequent analyses. Genes appearing to be exclusive to or shared (intersections) by the experimental groups were determined using the script PERL, and shown as Venn diagrams.

The genes from microarray data (adjusted p-value <0.01) were also applied in Cytoscape software (BinGO plugin) to determine the gene ontologies (GO). With this result, were considered all significant enrichment terms using a hypergeometric test (FDR<0.01) to obtain enrichment in Biological process (BP), Molecular function (MF) and Cellular component (CC).

### Transcription Factor and Transcription Regulators in the Enrichment Terms

All genes present in BC, MF, CC, from enrichment terms analysis, were used to construct regulatory networks using the Ingenuity Pathway Analysis (IPA) software.

The transcription factors and transcription regulators enrolled to construct the regulatory networks were identified and classified as to whether or not they were differentially expressed in the microarray data.

To test the correlation among these genes, androgen receptor (Ar) and the estrogen receptors (Esr1 and Esr1), we constructed a minimum spanning tree (MST) using the Pajek software. A correlation matrix was prepared using the values of relative expression for each gene extracted from the results published by Su and co-workers [22]. The correlation distance matrix was calculated using the expression 
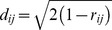
, where 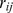 is the correlation coefficient between list of variables i and j.

The standard variation of the values obtained for the relative expression on the different tissues was used as vectors in the MST. A detailed description of this procedure and their applications will be published elsewhere.

### TF Binding Sites in the Proximal Promoter Region of the Most-regulated Genes

To find out more putative TF that can act in the prostate gland after androgen deprivation, it was selected the 20 most regulated genes (10-up and 10-down) exclusive to each treatment (Cas, E2 and Cas+E2) and shared by the three treatments according to fold-change from microarray data. In total, 80 genes were analyzed individually to determine putative TF binding sites in the 3,000 bp upstream of the gene transcription start. To run this analysis the selected sequences were loaded in the Match-1.0 program (www.gene-regulation.com), and the parameters (a) vertebrate matrices and (b) cutoff for the matrix of false-positive and false-negative groups were selected to identify the TF binding sequences.

The resulting list was sorted according to the total number of binding sites in the promoter regions. We also compared the number of binding sites for the identified TF with those in the proximal promoter region of the GAPDH and TBP-7 genes, which were selected as internal controls, because their mRNA levels were not affected by the treatments, as determined by qRT-PCR.

The TF density (number of binding sites in the promoter region divided by the number of genes) in the regulated genes was compared with the TF density in the promoter region of the internal control genes, as a way to eliminate those with a wide distribution in the genome. Finally, we identified the TF that were exclusive (or enriched) to each experimental group.

**Figure 1:**
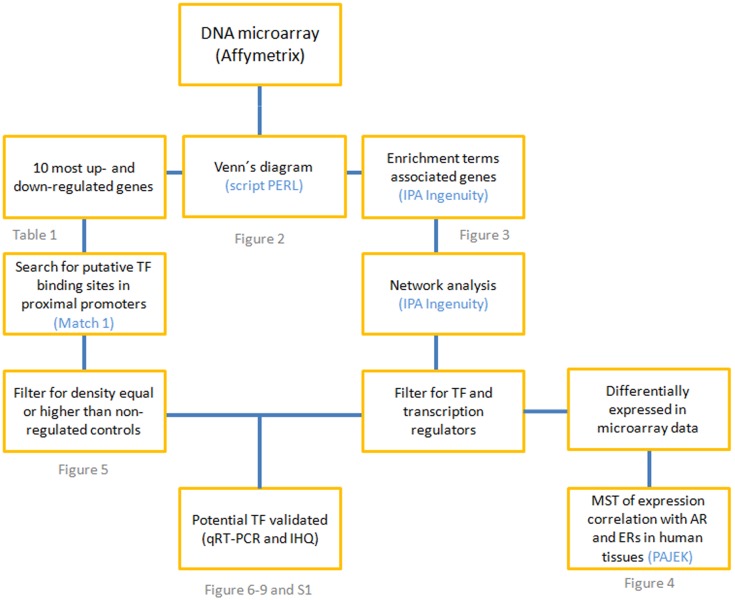
Summary of the sequential analyses performed in this study.

Figure 1 summarizes the combination of methodologies employed in this study.

### Quantitative Real-time PCR

Quantitative real-time PCR was used to determine the TF expression at the mRNA level. We followed the kinetics of the mRNA variation for up to seven days after castration. RNA samples were reverse-transcribed to cDNA by a Super Script III First-Strand Kit (Invitrogen, Carlsbad, CA, USA). cDNA was mixed with TaqMan Universal PCR Master Mix (Applied Biosystems, Foster City, CA) and inventoried assays Rn01756649_g1 (*Elk-1*), Rn01493436_m1 (*Evi-1*), Rn01750242_m1 (*Mybl2*), Rn01442895_m1 (*Mybl1*), Rn01399583_m1 (*Nfkb1*), Rn01413842_g1 (*Nfkb2*), Rn01502266_m1 (*Rela*), Rn00583735 (*Gata2*), Rn01648938_m1 (*Nfyb*) and Rn00573309_m1 (*Hnf4a*). For *Rel*(*cRel*), we designed the primers FW: 5′–CCGGCCGGACAGCTTT and Reverse: 5′–GCCAGCCCCGTCTAGGAA, and the probe: FAM-CTCTAACTCACAAGGTGTCCT. Our attempts to produce primers and probes for RelB produced no acceptable results, and therefore the evaluation of this TF was limited to immunofluorescence. The reactions were conducted and analyzed to determine the threshold cycle in an Applied Biosystems 7300 Real-Time PCR System. The results are presented as fold-changes calculated using the ΔΔCt with the use of *Gapdh* (Rn01775763_g1) as internal control, after testing 9 routinely used internal controls.

The statistical analyses included ANOVA and *post hoc* Tukey’s test, considering the results as significant when *p*<0.05.

### Immunofluorescence Analysis

The ventral prostates of control and castrated animals at day 3 after surgery were collected and immersed in Tissue-Tek O.C.T. Compound (Torrance, CA, USA) and frozen in liquid nitrogen. Five-µm cryosections were obtained and used for immunofluorescence. Sections were fixed first in cold methanol and then in 2% paraformaldehyde for 10 min each. The sections were permeabilized with 0.2% Tween 20 in PBS for 15 min at room temperature. The autofluorescence was quenched with 10% H_2_O_2_ in PBS for 15 min. Non-specific protein-protein interactions were blocked by incubation with 10% pre-immune serum in PBS for 1 h at room temperature.

The primary antibody against MYBL2 (goat, cat. ab53511) was obtained from ABCAM (Cambridge, MA, USA). Antibodies against EVI1 (rabbit, cat. 2593S), ELK1 (rabbit, cat. 9182S), GATA2 (rabbit, cat. 4595S), NFKB1 p105/p50 (rabbit, cat. 3035S), NFKB2 p100/p52 (rabbit, cat. 4882S), NFKB p65/RELA (rabbit, cat. 8242S), RELB (rabbit, cat. 4954S), and REL (c-Rel) (rabbit, cat. 4774S) were obtained from Cell Signaling (Beverly, MA, USA). They were diluted 1∶300 in the 10% pre-immune serum in PBS and incubated overnight at 4°C.

The tissue sections were rinsed three times in PBS and then incubated with either Alexafluor 546-conjugated donkey anti-goat Igs or goat-anti rabbit Igs (cat. A11056 and cat. A11010; Invitrogen) secondary antibodies diluted 1∶2000 in PBS for 1 h at room temperature. After incubation in the secondary antibody, the slides were washed in PBS and then incubated in DAPI for 10 min to stain the nuclei. Photo documentation and analysis were done using a Leica DM 2500 microscope equipped for fluorescence imaging.

## Results

### Differential Expression Profiles

**Figure 2:**
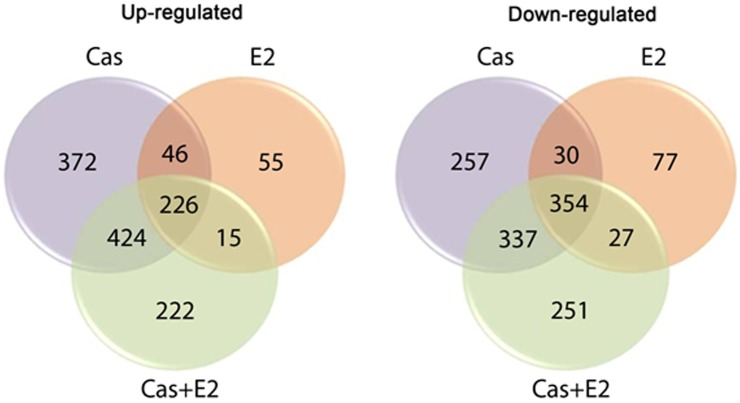
Venn diagrams showing the number of genes that were differentially expressed. The diagrams show the genes that are exclusive to each treatment and those that are shared by two or all three experimental groups (Cas, E2 and Cas+E2) compared to the control group.

Microarray analysis showed the gene expression profile of the treated groups compared with the control animals. The criteria of 1.5-fold variation and a p<0.01 value were used to select the genes with differential expression. This resulted in a total of 2,693 genes that showed differential expression compared to the controls. One-fifth (21.5%) of these genes (580 genes) were shared by the three experimental conditions employed in this study (226 up and 354 down), as shown in the Venn diagrams in Figure 2. In addition to sharing these differentially expressed genes, the Cas group showed the largest number of differentially expressed genes (2,046 genes), while the E2 group showed 830 genes, and only 132 exclusive genes. The combined treatment (Cas+E2 group) showed an intermediate number of differentially expressed genes (1,856 in total) with 473 exclusive genes. It became clear that androgen deprivation (Cas group) resulted in more exclusive genes that were up-regulated than those that were down-regulated, suggesting that the changes achieved by androgen deprivation require an active process of gene expression to coordinate the modifications associated with gland regression and tissue remodeling. In contrast, more genes were down-regulated in response to the E2 treatment or the combination of Cas+E2. Thought.

### TF in the Enrichment Terms

**Figure 3:**
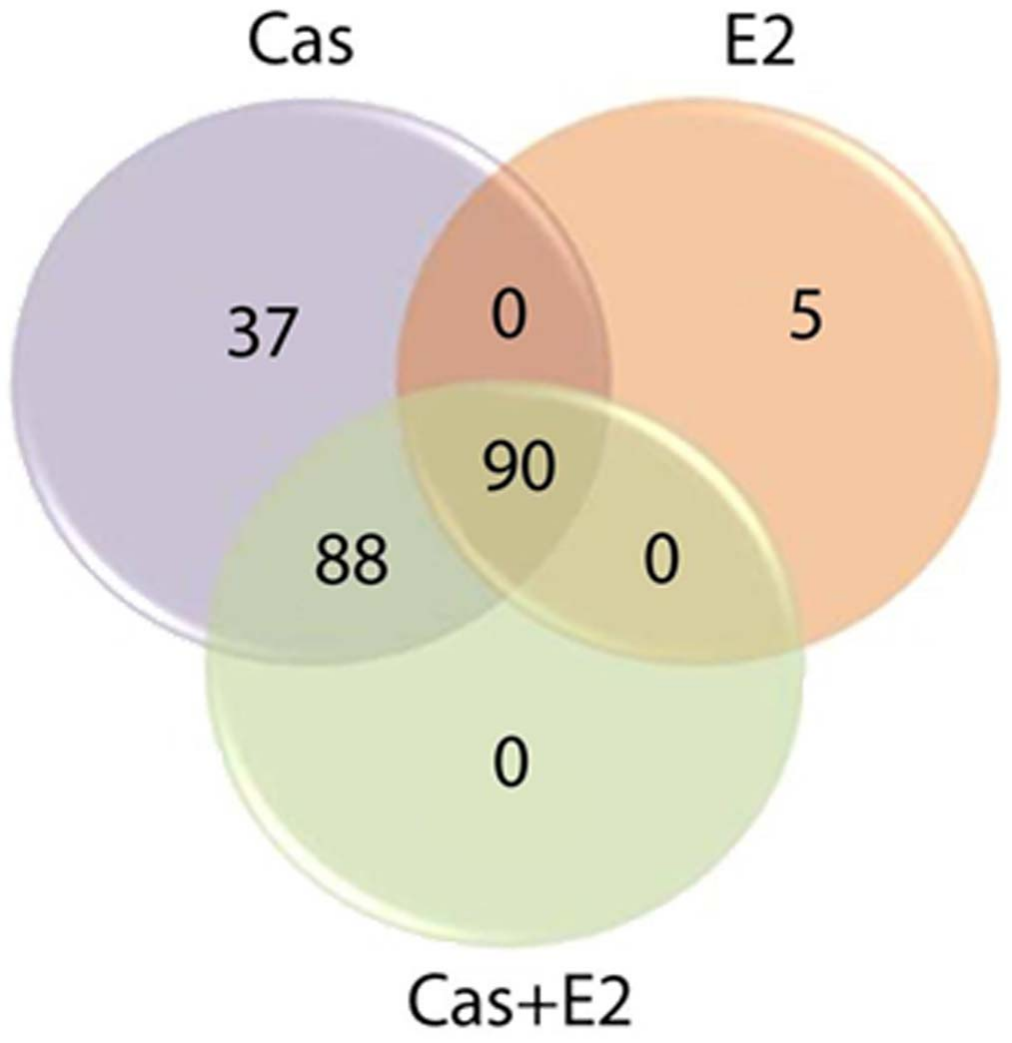
Number of enrichment terms exclusive or shared among groups. The three treatments shared 90 enrichment terms. The highest number of enrichment terms was found in the Cas group. The Cas+E2 group showed no exclusive enrichment term.

Enrichment terms were observed in the Cas (37 terms) and E2 (5 terms) groups, in the intersections between the three experimental groups (common genes) (90 terms), and in the intersection of the Cas and Cas+E2 groups (88 terms) (Fig. 3).

After the identification of the enrichment terms, we used the IPA Ingenuity software to identify gene interaction networks. Then, 135 transcription-regulation related candidates appearing in those networks were isolated. Shown in Table S1 are the candidates that were up- (40) or down-regulated (16) in the microarray results. The analyses clearly showed that more TF were up-regulated than down-regulated in each experimental condition. This might reflect the predominance of the androgen-regulated genes, and perhaps also the complexity of genes involved in the squamous metaplasia transition induced by estrogen stimulation (results not shown) [23].

**Figure 4:**
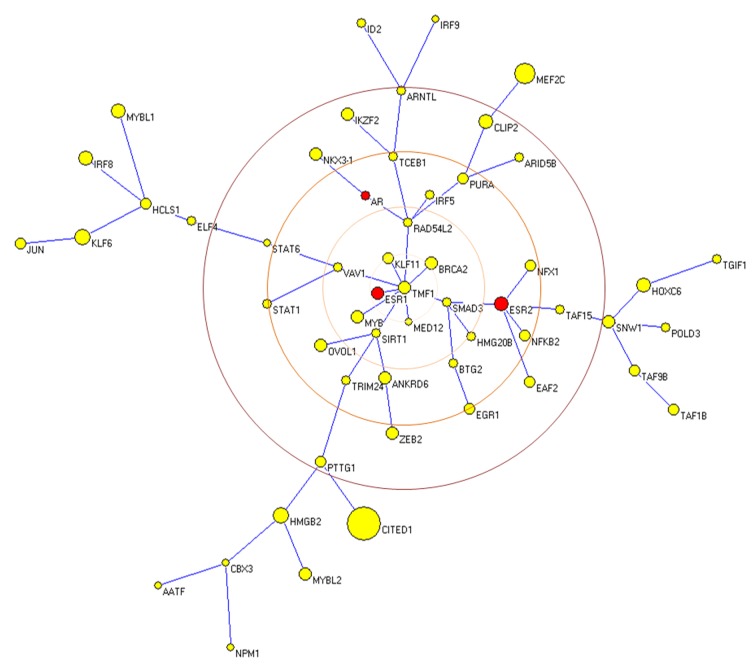
Differentially expressed TF and transcription regulation-related genes. Transcription regulation-related genes indentified by the IPA Ingenuity program and showing differential expression in those subgroups containing enrichment terms. Red and green colors highlight the up- or down-regulated genes as compared with the control group, respectively. A minimum spanning tree is used to show the correlation among the expression variation of the differentially expressed genes (yellow) plus the androgen receptor (AR) and estrogen receptors (ESR1 and ESR2) (red) in human tissues. Each gene is indicated as a vertices and their size represent the standard deviation of their fold changes in different tissues. The edge length is the distance calculated from the correlation matrix as explained in M&M. Of note, are the centrality of TMF1 and the short distances between the pairs AR-NKX3-1, ESR1-BRCA2, and ESR2-NFKB2.

We calculated the correlation among the expression of these selected genes and that for the *Ar*, *Esr1* and *Esr2* in a series of human tissues and opted to show the results as a MST, after calculating the distances among the expression profiles. The resulting MST is also shown in Figure 4. One obvious observation is that genes differentially expressed in the microarray data have different correlation with the *Ar*. TMF1 showed the highest centrality in the tree. It was interesting to note that *Ar*, *Esr1* and *Esr2* also occupied a relatively central position. The efficiency of this construction in showing the correlation among the expression pattern of different genes is manifested by the short distances between the pairs Nkx3.1/Ar; Esr1/BRCA2, and Esr2/NFkB2, because of well known functional associations among them.

### TF in the Promoter Region of the 10 Most up- or down-regulated Genes

From the microarray analysis, we selected the ten most up- or down-regulated genes, as compared to the controls, that were exclusive to each experimental group, and also those shared by the three treatments (Table 1).

**Table 1:** The eighty most regulated genes.

Groups	Up-regulated	Down-regulated
**Cas**	Ska3/LOC689296/LOC681325 Aspn/RGD1562462/Slamf9 Ifit1/Kcnd2/Rnase2/Rpl21	Ocm/Furin/Isyna1 LOC287167/Aox3/Dmd/Slc17a9 Tomm7/Tnnt2/Hspb6
**E2**	Ankrd11/N5/Hamp/Sft2d1 LOC691979/nucleolin/Tns4 Slc39a1/Dync2h1/LOC290071	Sparc/Slc25a24/Susd2/Lcn2 Ly6c/Pi16/Glrx1/Kcnmb1 C1H6orf35/Tmem47
**Cas+E2**	Sh3rf2/LOC310926/Macc1/Chi3l1/Krt15/LOC259246 Rnase1l1/Sdr16c5/Obp3/Prol1	Mrpl41/Sms/Defb50/Hectd2 MGC109340/Scnn1g/Me1 V1rd15/Twf1/Pmp22
**Shared**	RGD1565682/Cfh/Gnat2 Lrrc34/Mmp12/Gpnmb/Abcc3/V1rk2/Blnk/Mmp15	Ugt2b34/Cntn6/Cyp2c13 Oas1k/Neto2/Cpa6/Cdh12 Wfdc3/Lrrc37a/Ldoc1

The ten most up- or down-regulated genes that were exclusive to each of the experimental groups or were shared by the three treatments. These genes are involved in different functions (see Appendix 1 for details on the annotated functions for these genes).

For each of these 80 genes, we did a search for transcription-binding sites in the proximal 3,000-bp promoter region. The density of the TF binding sites in the promoter was calculated by dividing the number of binding sites by the number of genes considered (i.e. 80 regulated genes and 2 controls) compared to those found for the *Gapdh* and *Tbp7* genes. These two genes were chosen from a list of nine routinely used internal controls because their expression did not vary in the different experimental groups, as determined by qRT-PCR.

**Figure 5:**
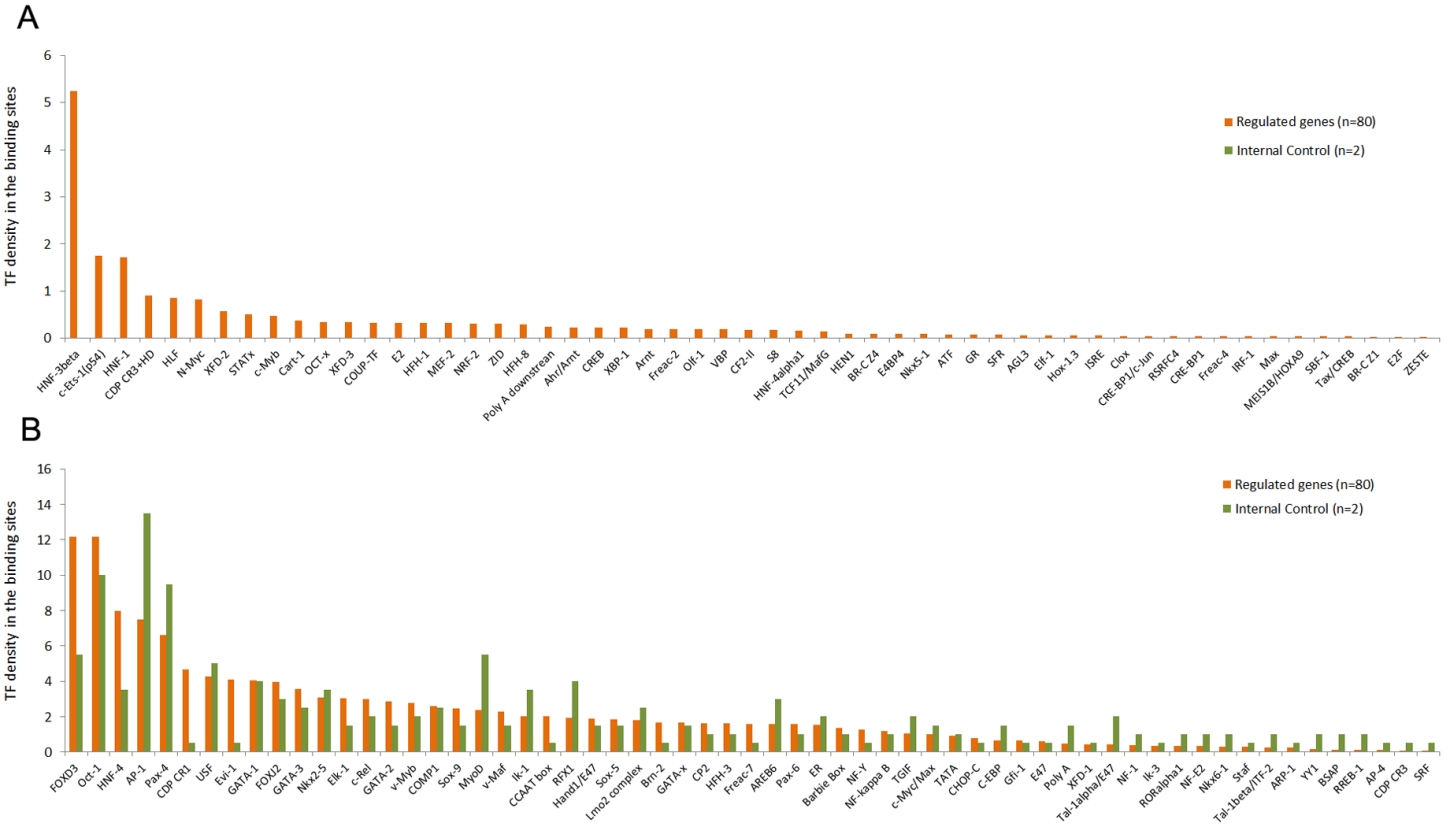
TF with possible binding sites found in the 80 most-regulated genes. (A) TF binding sites not found in the internal control genes. (B) TF binding sites also found in the proximal promoter of internal control genes. Density values≤0.01 were omitted.

We found 55 putative TF binding sites that were absent from the promoter region of the control genes *Gapdh* and *Tbp7* (Fig. 5A). Most of these TF binding sites were found in very low density. Figure 5B shows the 61 TF binding sites found in the promoter region of the regulated genes and in the promoter region of the controls.

Finally, we compared the list of TF appearing in the gene regulatory networks obtained with the use of Ingenuity (135 candidates) and those TF whose putative binding sites were found in the proximal promoter of the 10 most-regulated genes that were equal to or higher than in the promoter region of the non-regulated genes (*Gapdh* and *Tbp7*) (84 candidates). Eight transcription factors were present in both lists: EVI1, NFY, HNF4, ELK1, GATA2, REL (c-Rel), MYB and NFκB. MYBL1 (A-MYB) appeared up-regulated in the microarray data and was chosen along with its family member MYBL2 (B-MYB) for further analysis. Given the incompleteness of information about the expression of NFkB family members in the prostate, we selected different members for further validation. Unexpectedly and perhaps fortuitously, this approach excluded important TF, such as OCT1 [5], AR and estrogen receptors.

### RT-PCR Validation of TF Expression

We validated the expression of the eight TF, their variants, and some family members. Only the *Hnf4* gene was not found to be expressed in the adult prostate. The variation in expression of the remaining seven TF was studied for up to seven days after castration. We grouped them in three groups. *Mybl1*, *Mybl2* (members of the MYB family) and *Gata2* were grouped together because of their known expression in immature hematopoietic cells [24]–[26]. The second group included members of the NFκB family: *Nfκb1*, *Nfκb2*, *Rela* and *Rel* (*cRel*). The third group included the independent TF *Evi1*, *Elk1* and *Nfyb*.

**Figure 6:**
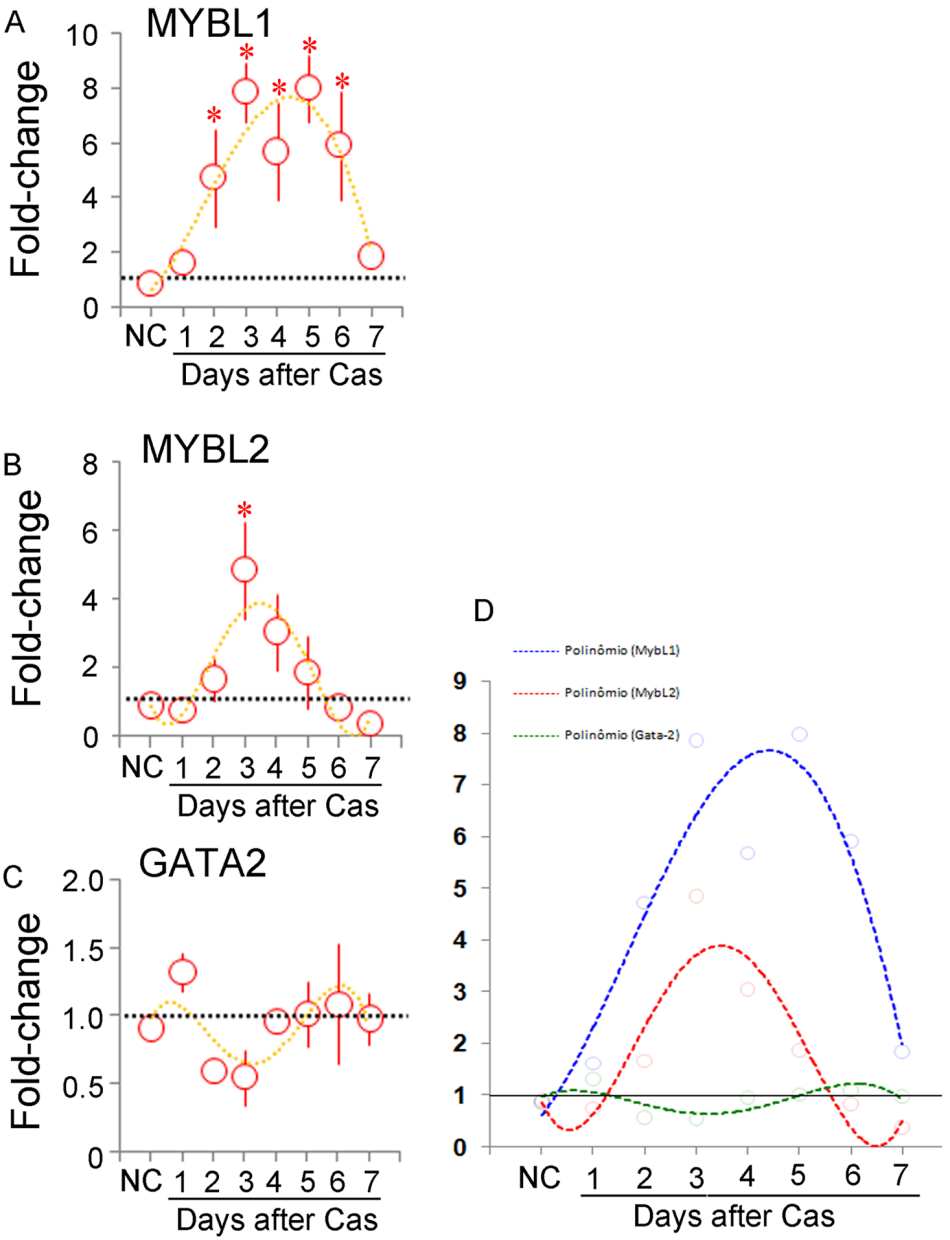
Kinetics of *Mybl1*, *Mybl2* and *Gata2* expression. Quantitative RT-PCR determination of the mRNA content for the *Mybl1* (A), *Mybl2* (B) and *Gata2* (C) genes in the prostate of non-castrated (NC) and castrated rats up to 7 days after surgery. The fold-change variation with respect to the controls is shown as the mean ± the standard variation (n = 3 for each time point). The asterisks indicate p<0.05. The dotted lines in each figure correspond to the fourth-power exponential fitting curve, and are shown together for the sake of direct comparison in D.

*Mybl1* and *Mybl2* showed statistically significant fold-increases after castration (Fig. 6A and B). While the latter showed less variation with a peak at day 3, the former increased as early as day 2. However, both genes increased only transiently. On the other hand, *Gata2* did not show significant variation in response to castration (Fig. 6C), in agreement with a previous notion that this gene is not affected by androgens, although it is known to coordinate the expression of AR-regulated genes [5], [27]. Direct comparison of the trend line obtained by the fourth-order polynomial (Fig. 6D) indicated that the oscillation in *Gata2* expression, though not significant, appeared complementary to that shown by *Mybl2.* This form of representation also revealed that *Mybl1* expression precedes and extends longer than that of Mybl2, and might be an inherent characteristic of their expression that warrants further investigation.

**Figure 7:**
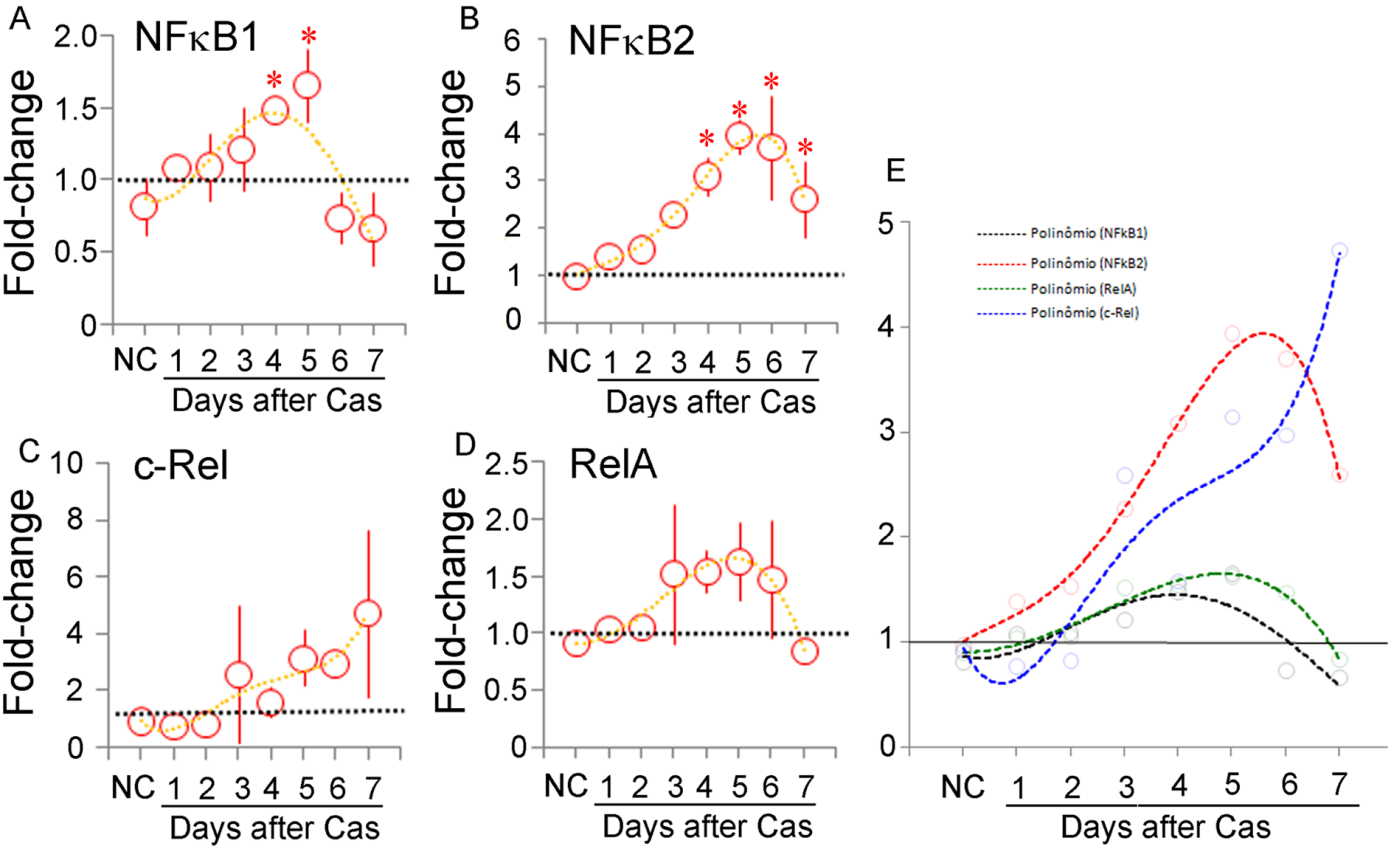
Kinetics of *Nfkb1*, *Nfkb2, Rel* and *RelA* expression. Quantitative RT-PCR determination of the mRNA content for the *Nfkb1* (A), *Nfkb2* (B), *Rel (c-Rel)* (C) and *RelA* (D) genes in the prostate of non-castrated (NC) and castrated rats up to 7 days after surgery. The fold-change variation with respect to the controls is shown as the mean ± standard variation (n = 3 for each time point). The asterisks indicate p<0.05. The dotted lines in each figure correspond to the fourth-power exponential fitting curve, and are shown together for the sake of direct comparison in E.

Statistically significant increases in fold-change were observed for *Nfkb1* (Fig. 7A) and *Nfkb2* (Fig. 7B) after castration, but not for *Rel* (*cRel*) (Fig. 7C) and *Rela* (Fig. 7D). *Nfkb1* and *Nfkb2* expression appeared transient, with peaks at days 4–5. Using the same direct comparison of the trend lines for the four genes together (Fig. 7E), and examining the non-significant variation measured for the *Rel* and *Rela*, we noted that *Rel* differed from the others in showing incremental increases within the timeline of the experiments, while the variation in the others seemed to be transitory. We speculate that the transient nature of gene expression observed for three of the NFKB members might be connected to transitory events in the adjustment of the castration-resistant cells to the androgen-deprived condition.

**Figure 8:**
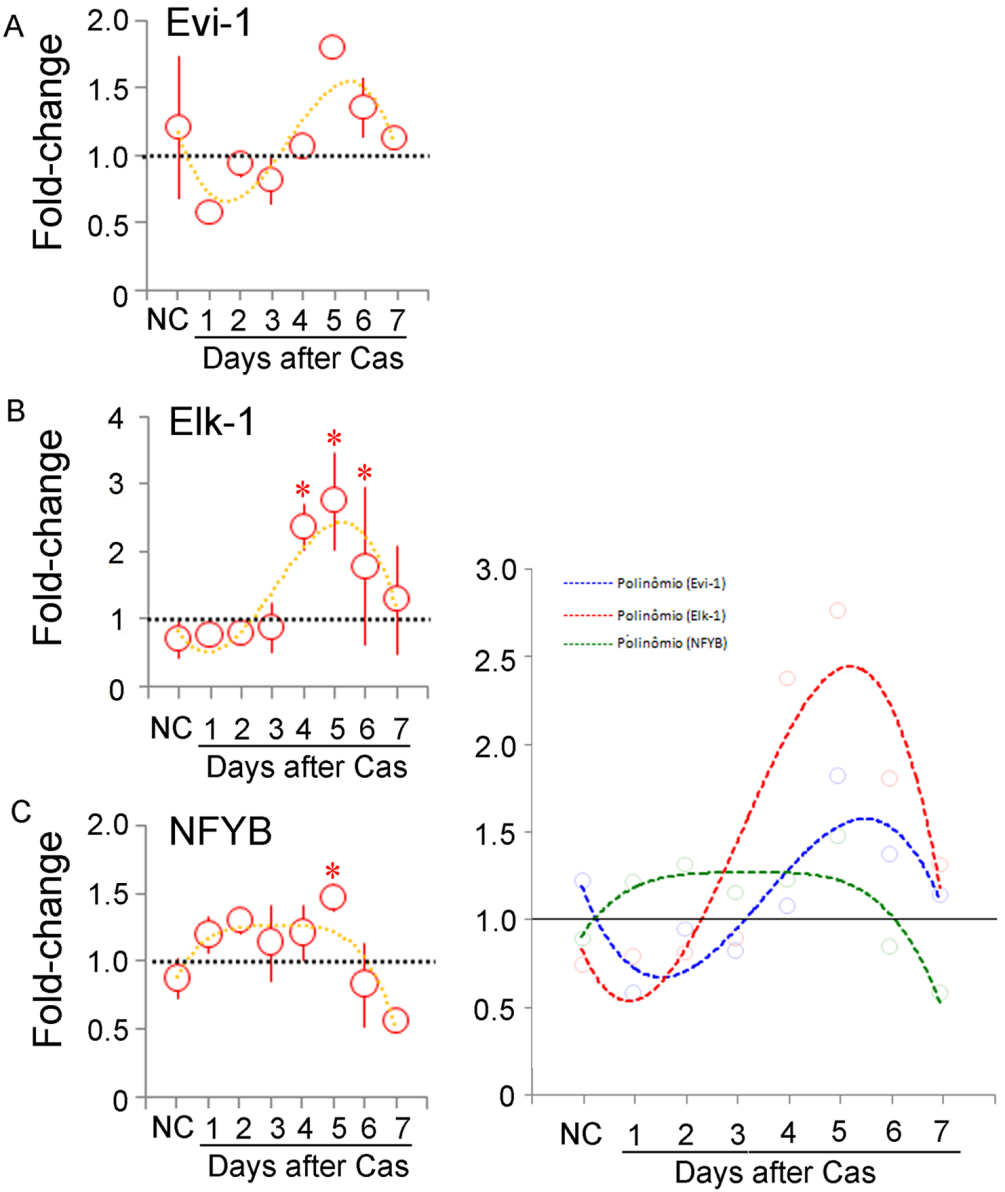
Kinetics of *Evi1*, *Elk1* and *Nfyb* expression. Quantitative RT-PCR determination of the mRNA content for the *Evi1* (A), *Elk1* (B) and *Nfyb* (C) genes in the prostate of non-castrated (NC) and castrated rats up to 7 days after surgery. The fold-change variation with respect to the controls is shown as the mean ± standard variation (n = 3 for each time point). The asterisks indicate p<0.05. The dotted lines in each figure correspond to the fourth-power exponential fitting curve, and are shown together for the sake of direct comparison in D.

The quantification of *Evi-1* expression in response to castration revealed no significant variation (Fig. 8A). In contrast, the quantification of *Elk-1* revealed not only a significantly increased expression, but also an interesting pattern, remaining steady for up to day 3 and then increasing up to 3-fold, before returning to the level of the control (Fig. 8B). *Nfyb* quantification and kinetics were equally interesting (Fig. 8C). Although a significant increase was only found at day 5, it showed a long lasting slight increase in mRNA content. Superimposing the trend lines (Fig. 8D) of these three apparently independent TFs provided no further elucidation of a stereotyped behavior, except for reinforcing the delayed increase in *Elk-1* expression after castration.

### Immunofluorescence Localization of the Newly Identified TF

**Figure 9:**
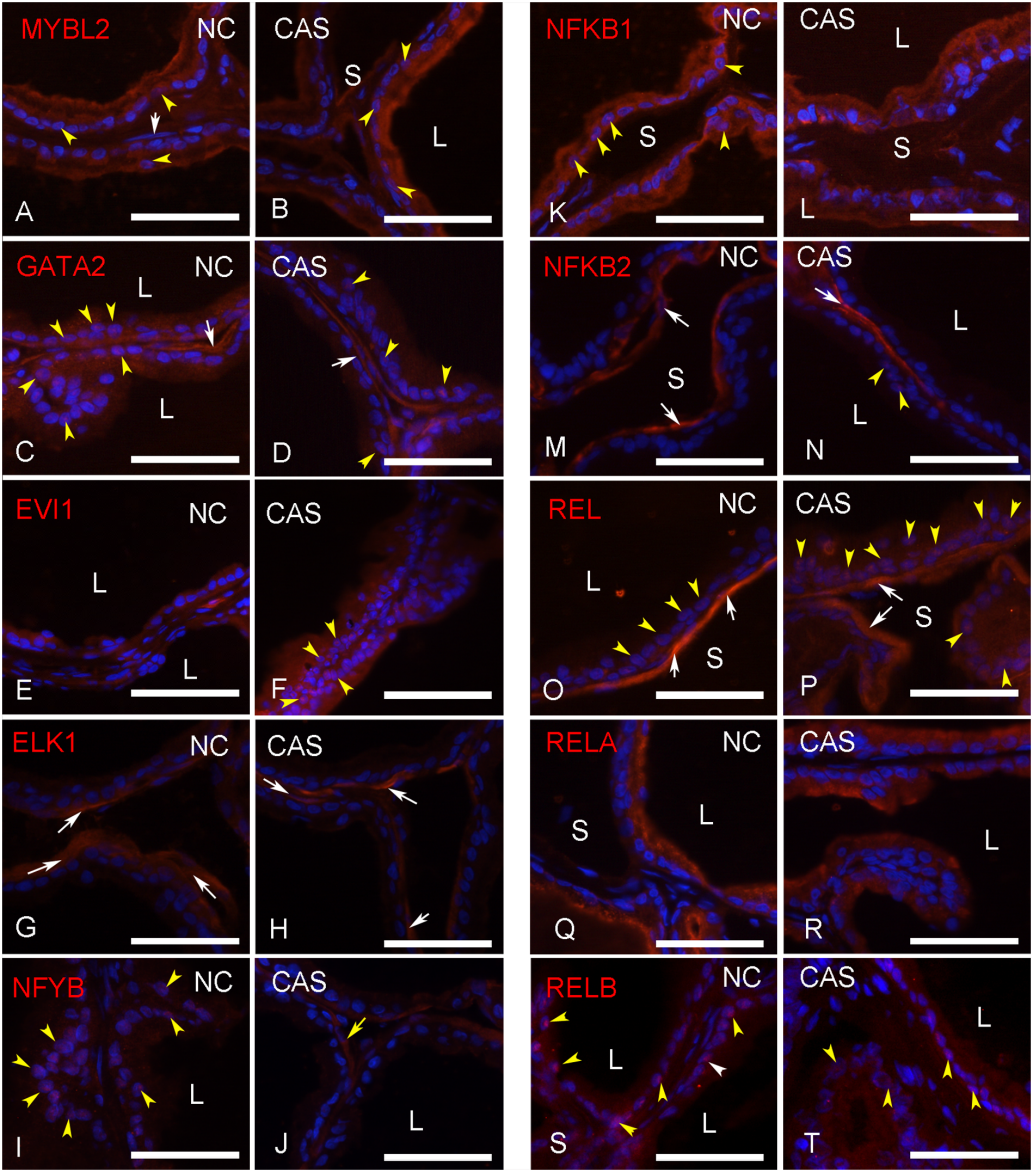
TF localization in epithelial and stroma cells. Immunohistochemical localization of selected TF (red) as indicated in the prostate of non-castrated controls (NC) and castrated rats 3 days after surgery (CAS). MYBL2 (A,B), GATA2 (C,D), EVI1 (E,F), ELK1 (G,H), NFYB (I,J), NFKB1 (K,L), NFKB2 (M,N), REL (O,P), RELA (Q,R), RELB (S,T). Nuclei were stained with DAPI (blue). White arrows indicate smooth-muscle cells. Yellow arrowheads indicate cells showing nuclear location of the TF. L  =  gland lumen; S  =  stroma. Scale bars = 50 µm.

The immunofluorescence revealed that the selected TF were differentially distributed in the epithelial and stromal cells of the rat ventral prostate (Fig. 9). Several TF are present in both epithelial and stromal cells, but had unique distributions (Fig. 9).

We found MYBL2 to be concentrated in the epithelial cells and discretely in the cell nucleus. GATA2, on the other hand, was found in both epithelial and stromal cells, with clear nuclear localization in non-castrated animals. The number of cells showing nuclear localization was reduced in animals examined 3 days after castration.

EVI1 was found in both epithelium and stroma, and showed nuclear localization in the epithelium after castration. ELK1 showed a restricted stromal localization in morphologically recognized smooth-muscle cells. NFYB was detected in the nucleus of epithelial cells in non-castrated animals. The nuclear localization was partly lost after castration. Stromal cells were also positive for NFYB.

NFkB1 was found in both epithelium and stroma in non-castrated and castrated animals. The epithelium also expressed RelA and RelB, which, similarly to NFkB1, were not responsive to castration in terms of epithelial/stromal distribution. In contrast, REL was expressed in the epithelium after castration and was found in the cell nucleus. Since nuclear translocation of this (and other family members) occurs upon activation [28], this location is a good indication that REL is activated after castration. NFkB2 and REL were found exclusively in stromal cells, and maintained this distribution after castration. In contrast, the expression of RelB in the stroma was suppressed in response to androgen deprivation. Figure S1 summarizes these findings.

## Discussion

By comparing the gene expression profiles in the prostate under different hormonal conditions (androgen deprivation, high dose of 17β-estradiol, and a combination of these), we identified seven TF (and some variants), which were further characterized with respect to their expression in the prostate and possible variations in response to castration. These TF were found to be differentially expressed and were grouped in three functionally important groups: (i) coordinators of the immaturity state or regulators of epithelial differentiation (MYBL1/MYBL2 and GATA-2), (ii) coordinators of the ability of the organ to function as an immune barrier (NFκB family members), or (iii) early-response genes or genes related to the response to extracellular signaling (EVI1, ELK1 and NFYB), which together coordinate the epithelial and/or stromal cell behavior under androgen-deprived conditions.

It is well understood that treatment with androgens increases the AR and coactivator occupancy in the PSA enhancer and promoter regions [29]–[33]. Although the regulation of genes such as PSA by the AR in prostate cells seems to be relatively simple, and the prostate weight loss after castration is a direct effect, complex mechanisms are involved in the adaptation of the gland to varying levels of androgen stimulation.

We considered that extracting information from the mass of genes revealed by the present experiments using the usual tools could be misleading, in the sense that some regulatory molecules might show subtle or no variations in mRNA content and might escape detection in assays of differential expression. Therefore, we defined an experimental approach to filter for druggable TF that could function as state-defining regulatory hubs by orchestrating different functions related to tissue remodeling and as regulators of differentiation, among others, and localizing these TF to either the epithelium and/or the stroma, and employed a MST to determine the correlation among their expression levels is a number of tissues.

We identified TF genes in regulatory networks established from the enrichment terms and from the analysis of the proximal promoter region of the genes that were regulated by androgen deprivation, as revealed by the microarray analysis and referenced not only to the normal androgen levels in non-castrated rats, but also to the high-dose estrogen stimulus.

The search for biological relationship between the TF found in the selected networks and the steroid hormone receptors *Ar*, *Esr1* and *Esr2* was unveiled by calculating their expression correlation (and the resulting distance matrix) and constructing a MST. One obvious observation is that genes differentially expressed in the microarray data have different correlation with the *Ar*. One surprising finding was the high centrality of TMF1, named after TATA element modulatory factor [34], and later characterized as the first androgen receptor coactivator [35]. This factor was later characterized as a golgin and found in both nuclear and cytosolic factors [36], [37]. This dual localization and functional properties is intriguing and will certainly deserve further research. The MST also demonstrated that *Ar*, *Esr1* and *Esr2* also occupied a relatively central position. Nonetheless, the efficiency of this construction in showing the correlation among the expression pattern of different genes is manifested by the short distances between the pairs Nkx3.1/Ar; Esr1/BRCA2, and Esr2/NFkB2, because of well known functional associations among them.

Comparison of these TF with those whose putative binding sites in the promoter regions of the regulated genes uncovered a list of interesting candidates: EVI1 (Mecom), NFY, HNF4alpha, ELK1, GATA2, MYB, REL and NFkB. These eight transcription factors by the *in silico* analyses were validated, and only HNF-4 was found not expressed in the prostate (positive PCR and immunohistochemistry in the liver, used as positive control; data not shown).

### MYBL1, MYBL2 and GATA-2 Define Lineage Commitment and the Immaturity State of Epithelial Cells

The genes *Mybl1* and *Mybl2* belong to the MYB family, along with c-MYB. Both of them showed significantly enhanced expression under androgen deprivation. Besides, these TF have multiple binding sites in the promoter region of the regulated genes, and were found in enrichment gene networks in the Cas and Cas+E2 (data not shown).

MYBL2 is related to proliferative activity. Use of the MMTV-PyMT mouse was instrumental in the discovery that Myb is essential for the development of mammary tumors, but not ultimately for their progression [38]. *In vitro* tests showed that normal mammary epithelial NMuMG cells transduced with Myb show high proliferation and reduced apoptosis [39], consistent with Myb activation during the S through G2/M phases of the cell division cycle [40]–[42]. MYBL2 was reported to be associated with the control of expression of the anti-apoptotic protein bcl-2, by physically interacting with the promoter of this gene [43].

Recently, the functional aspects of *Myb* expression were investigated in LNCaP and its derivative C4-2 cells after super-expression or knockdown experiments [44], after the realization that *Myb* is frequently amplified in CRPC [45]. The authors provided solid evidence that *Myb* affects proliferation, adhesion, anchorage dependency, and several epithelial to mesenchymal transitions (EMT), and proposed that *Myb* is closely associated with disease progression [44].

It remains to be determined what the direct targets of MYBL1 and MYBL2 in the prostate gland are, and how these transcription factors interact with AR in the response to variations in androgen levels.

On the other hand, GATA-2 has more-evident effects on prostate cancer cells. GATA-2 and Oct-1 were found to be sequentially recruited to the enhancer region of AR-regulated genes [5]. Nonetheless, GATA-2 was further characterized as a factor in high risk of recurrence, PSA recurrence, and metastatic progression. Notably, the high expression was not associated with any detectable mutation in GATA-2 in these tumors [27]. The authors also demonstrated that GATA-2 regulates KLK2, but not AZGP1, two genes that are directly regulated by AR.

One study has demonstrated that GATA-2 is necessary for non-small cell lung cancer, via regulation of the proteasome, IL-1/NFκB signaling and Rho-signaling pathways, thereby providing enhanced proliferation for RAS-mutation. The authors suggested that GATA-2 operates orthogonally to oncogenes, as it is not directly affected by the receptor tyrosine kinase/RAS pathway, and that the GATA-2-centered network could represent a stress-response mechanism. In line with this evidence, our results revealed no significant variation in *Gata2* expression after castration (Fig. 6C), indicating a relative independence from AR signaling. Finally, the presence of GATA2 in the cell nucleus (Fig. 9 and Fig. S1) indicates that GATA-2 is found in its active form.

To our knowledge, this is the first demonstration that MYBL1/MYBL2 and GATA-2 are expressed and likely involved in modulating the response to androgen deprivation in physiological, non-tumor conditions in prostate epithelial cells.

We are convinced that the immaturity state coordinated by MYB1/2 and GATA2 is a hallmark of epithelial cells that facilitates the transition to CRPC in humans and represent targets for a therapeutic approach to this devastating disease.

### NFκB Family Members Integrate the Epithelial and Stromal Functions as an Immune Barrier

Immune-system cells are found in the prostate, and are present in larger numbers after castration. Mast cells are readily identified by their characteristic morphology, and macrophages and lymphocytes have been reported by others [15]. It is also known that androgens regulate a specific immune/inflammatory response [15], [19], but in transcriptome analysis, the presence of immune cells influences or obscures the contribution of immune-response genes.

The combined strategy employed in this study allowed us to identify c-Rel and NFκB TF among those selected by bioinformatics, and to extend their characterization to other family members. The variations in gene expression and location in the epithelial and/or stromal compartments, in particular the appearance of REL in the epithelium and the disappearance of RelB expression in the stroma in response to castration, suggest a dynamic adaptation of these important TF to the absence of androgen stimulation, in particular the possible dimers that they can form, particularly after the silencing of the secretory function, the major role of the gland. Furthermore, the nuclear location of REL suggests that it is present in the active form and it may have a role in non-pathogen-induced functions of members of the NFκB, such as a blocker of apoptosis [46], and in the possible survival of epithelial cells in the androgen-poor environment.

Maldonado and collaborators have characterized the capacity of prostate stromal cells to recognize and respond to pathogen-associated molecular patterns (PAMPs), given the characteristic pattern of Toll-like receptor 4 (TLR4) expression [47]. They also extended the observations to determine the effects of castration, and showed that RELA (therein referred to as NFkB/p65), in combination with CD14 and MyD88, coordinates the adaptation of epithelial and smooth-muscle cells to androgen deprivation, including the ability to produce surfactant protein D (SP-D) [48], [49].

The results reported here are in good agreement with these functional assays. They add an extra layer to the mechanisms regulating the ability of the prostate to deal with infectious agents, and reinforce the notion that the epithelium and stroma perform complementary and perhaps overlapping functions as an immune barrier. It will be interesting to determine how these functions correlate with the gland function to produce and secrete anti-inflammatory factors, as well as their variations and function in human prostatitis.

Nonetheless, we suggest that the combined functions of these TF regulate the recruitment of immune cells to the organ and also the functioning of these immune cells, including the ability to concentrate immunoglobulins (mostly IgA and IgG) in the secretion.

### Immediate Early-response Genes and NFYB

The EVI1 (Mecom) function was found to overlap with the FOS early-response gene [50]. Elk-1 is among the TCF factors that regulate the cell response to extracellular signals, and is a direct target of MAP kinase/ERK1 phosphorylation to control the expression of c-fos [51], [52]. ELK-1 distribution was restricted to the smooth-muscle cells and showed increased expression after four days under androgen deprivation, while EVI1 and NFYB were found in both the epithelium and the stroma, and were irresponsive to androgen. This is compatible with the changes in these cells in the first week after castration, most obviously a reduction in cell volume [53], and with a reported involvement with the reorganization of collagen fibers in the extracellular matrix [54]. ELK1, Serum Response Factor (SRF) and NFκB (NFKB1/p50 and RELA/p65) show increased expression in the response of vascular smooth-muscle cells to oxidative stress caused by hemin [55]. Yeh et al. (2009) found that increased expression of ELK1 with increased tumor grading in prostate cancer and NFYB are among the regulatory networks that differ in cancer cells as compared to normal tissue [20]. However, direct regulation of ELK1 by the AR [56] and estrogen-receptor [57] pathways has been suggested.

NFYB forms ternary complexes with NFYA and NFYC. NFYB shows interaction with p53, with significant overlapping target genes in HCT116 cells [58], with emphasis on ER-stress regulators and regulators of p53 itself. Notably, NFYB interacts with p53 to downregulate Chk2, which is associated with DNA damage-induced cell cycle progression in G2, and specifically targets cdc25 and p53 [59], and p73 to block the PDGFB receptor [60]. It will be interesting to investigate how NFY is involved in the reported dissociation of p53 and apoptosis in prostate regression after castration [61].

Taken together, the present data reveal novel TF that may contribute not only to prostate carcinogenesis and progression to CRPC, but also to the regulation of gene expression during the physiological adjustment of this organ to androgen variations in seasonal reproducers or in social groups with a male hierarchy.

## Supporting Information

Figure S1 **TF localization in epithelial and stroma cells.** Schematic drawing showing the distribution of the newly identified TF in the epithelium and stromal cells of the rat ventral prostate, as observed by immunohistochemistry, and their variations in response to castration at day 3 after surgery.
(TIF)
