# Supplementary figures and images for: Transcription Factors Involved in Prostate Gland Adaptation to Androgen Deprivation
Source: PLoS One. 2014 Jun 2;9(6):e97080. doi: 10.1371/journal.pone.0097080 (PMC4041569; doi:10.1371/journal.pone.0097080)

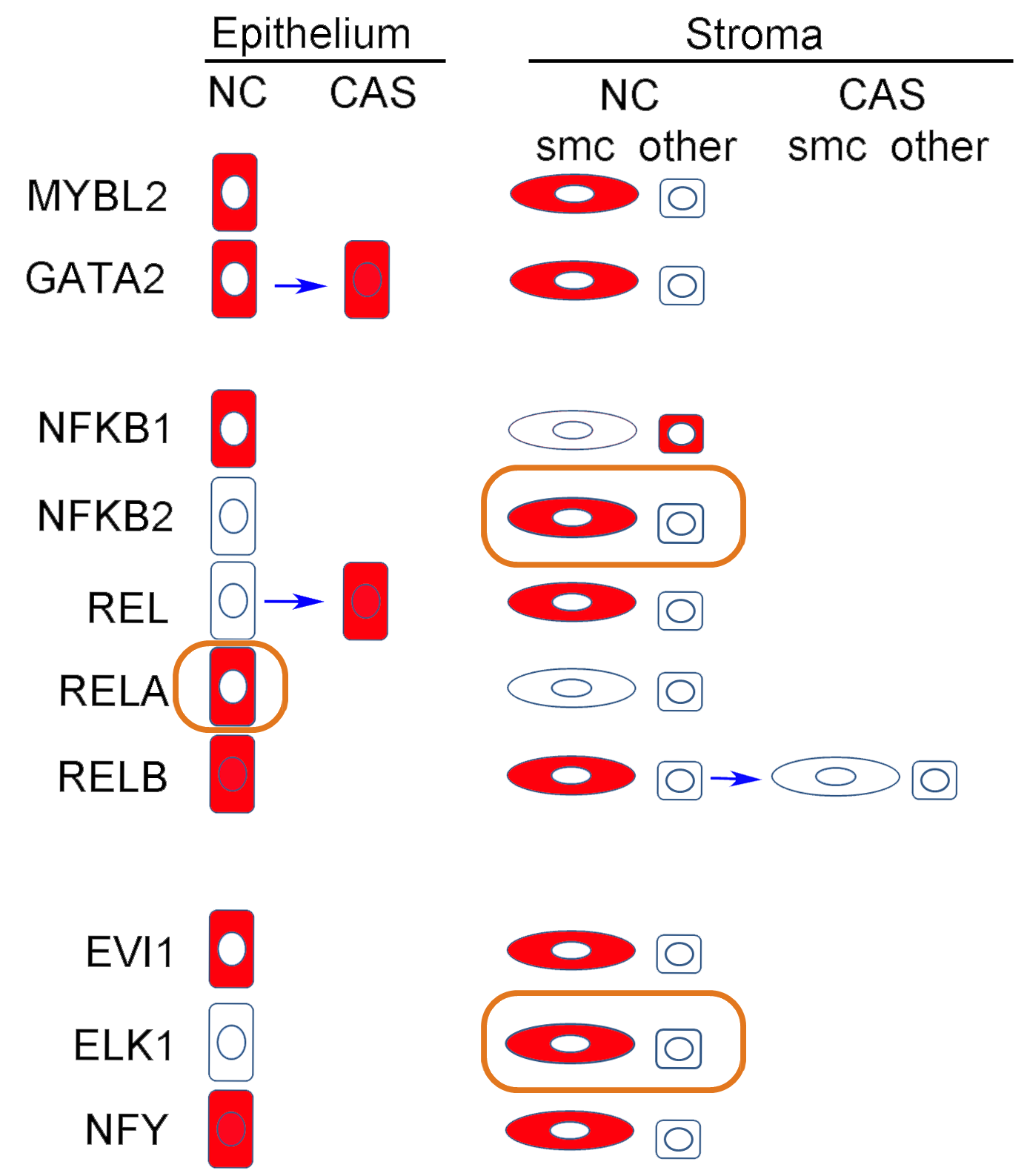

Supplement: Figure S1 — TF localization in epithelial and stroma cells. Schematic drawing showing the distribution of the newly identified TF in the epithelium and stromal cells of the rat ventral prostate, as observed by immunohistochemistry, and their variations in response to castration at day 3 after surgery. (TIF) [file pone.0097080.s001.tif]
